# Supplementary figures and images for: A role for worm cutl-24 in background- and parent-of-origin-dependent ER stress resistance
Source: BMC Genomics. 2022 Dec 20;23:842. doi: 10.1186/s12864-022-09063-w (PMC9764823; doi:10.1186/s12864-022-09063-w)

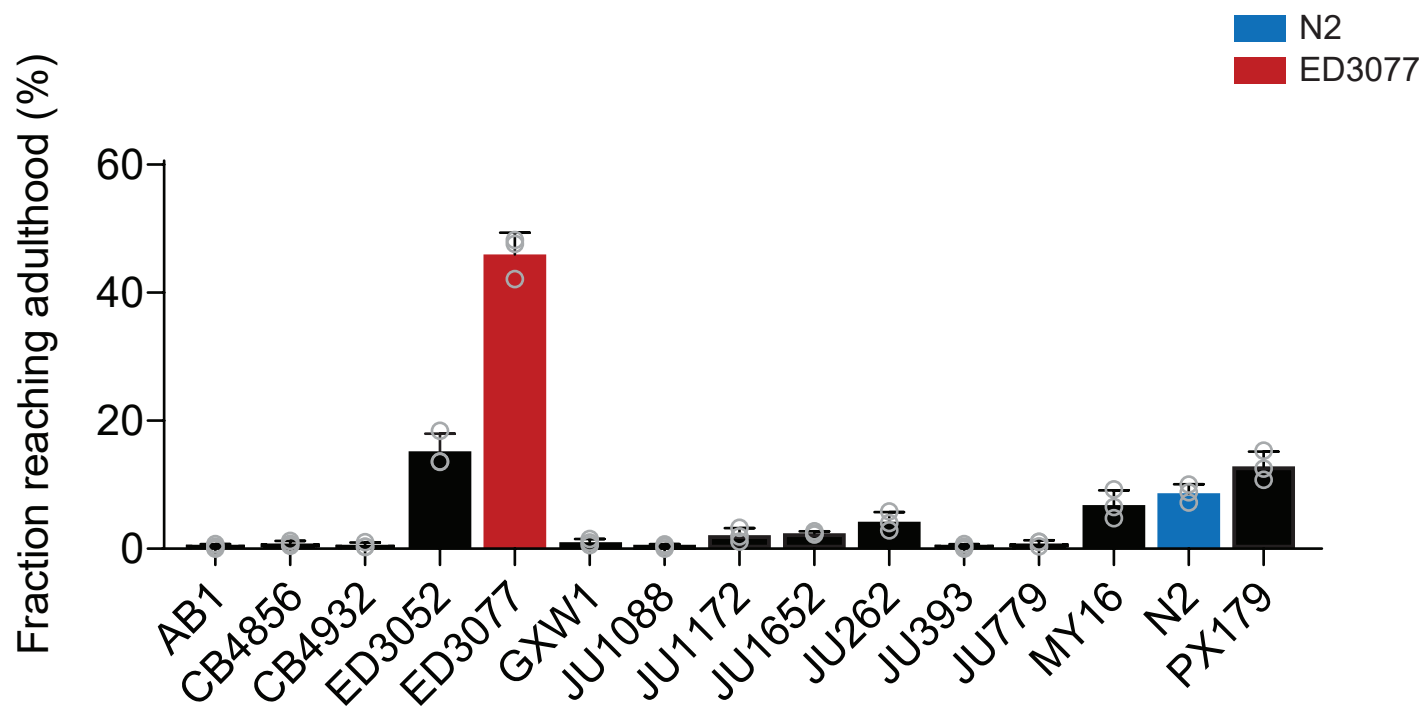

Figure S1

Supplement: Supplementary file 1 — Additional file 1: Supplementary Figure 1. Tunicamycin resistance phenotypes of wild C. elegans isolates. Data are as in Fig. 1 of the main text, except that all measures are reported as raw proportions without normalization. [file 12864_2022_9063_MOESM1_ESM.pdf]

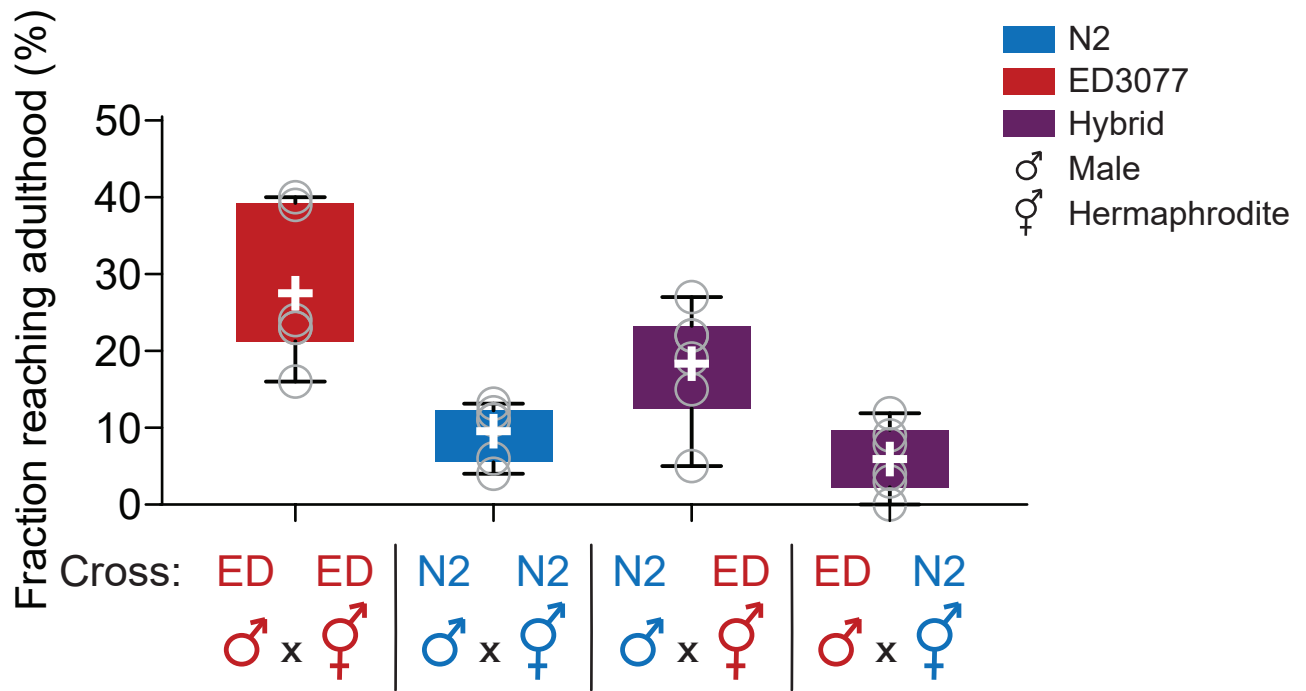

Figure S2

Supplement: Supplementary file 2 — Additional file 2: Supplementary Figure 2. Tunicamycin resistance of inter-strain hybrids depends on the parent of origin. Data are as in Fig. 2 of the main text, except that all measures are reported as raw proportions without normalization. [file 12864_2022_9063_MOESM2_ESM.pdf]

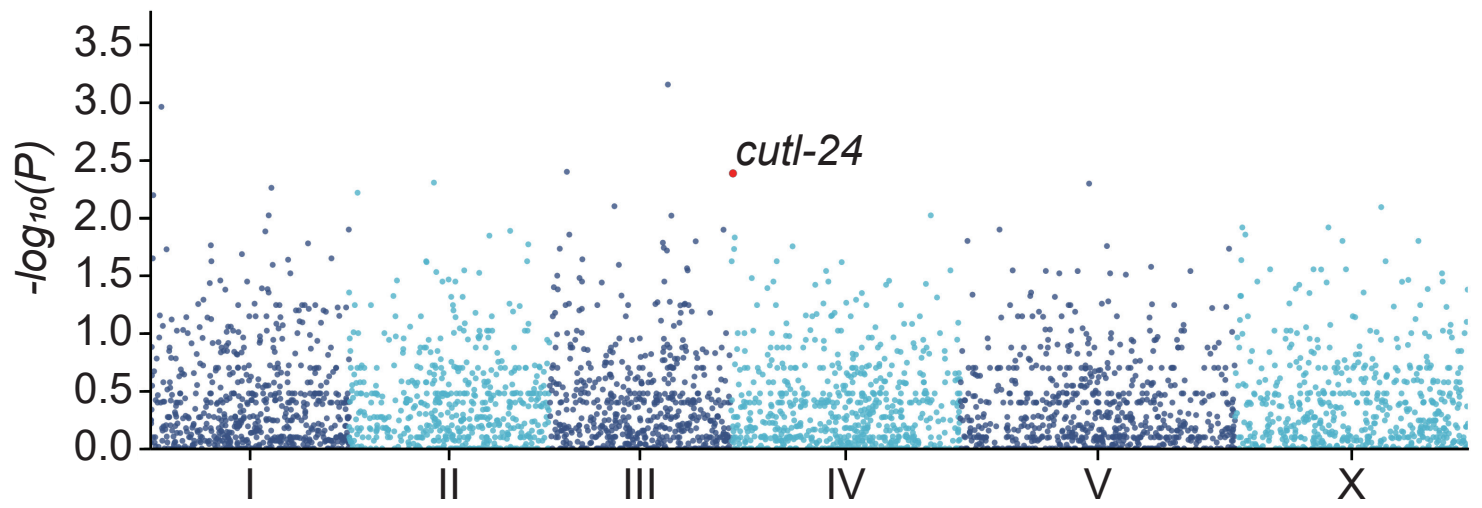

Figure S3

Supplement: Supplementary file 3 — Additional file 3: Supplementary Figure 3. Genomic distribution of significance measures from RH-seq mapping of tunicamycin resistance. Each point reports results from a reciprocal hemizygosity test of the impact, at one gene, of variation between ED3077 and N2 on development of their F1 hybrid in the presence of tunicamycin. The x-axis reports genome position of the respective gene, and the y-axis reports the negative log10 of the p-value from a Mann-Whitney test comparing two sets of sequencing-based measurements of hybrid strain abundance after development in tunicamycin: those from hybrid hemizygotes bearing a disruption in the ED3077 allele of the gene, uncovering the N2 allele, and those from hemizygotes bearing a disruption in the N2 allele (see Methods). Results for the focal gene of this study, cutl-24, are denoted in red. [file 12864_2022_9063_MOESM3_ESM.pdf]

S5a

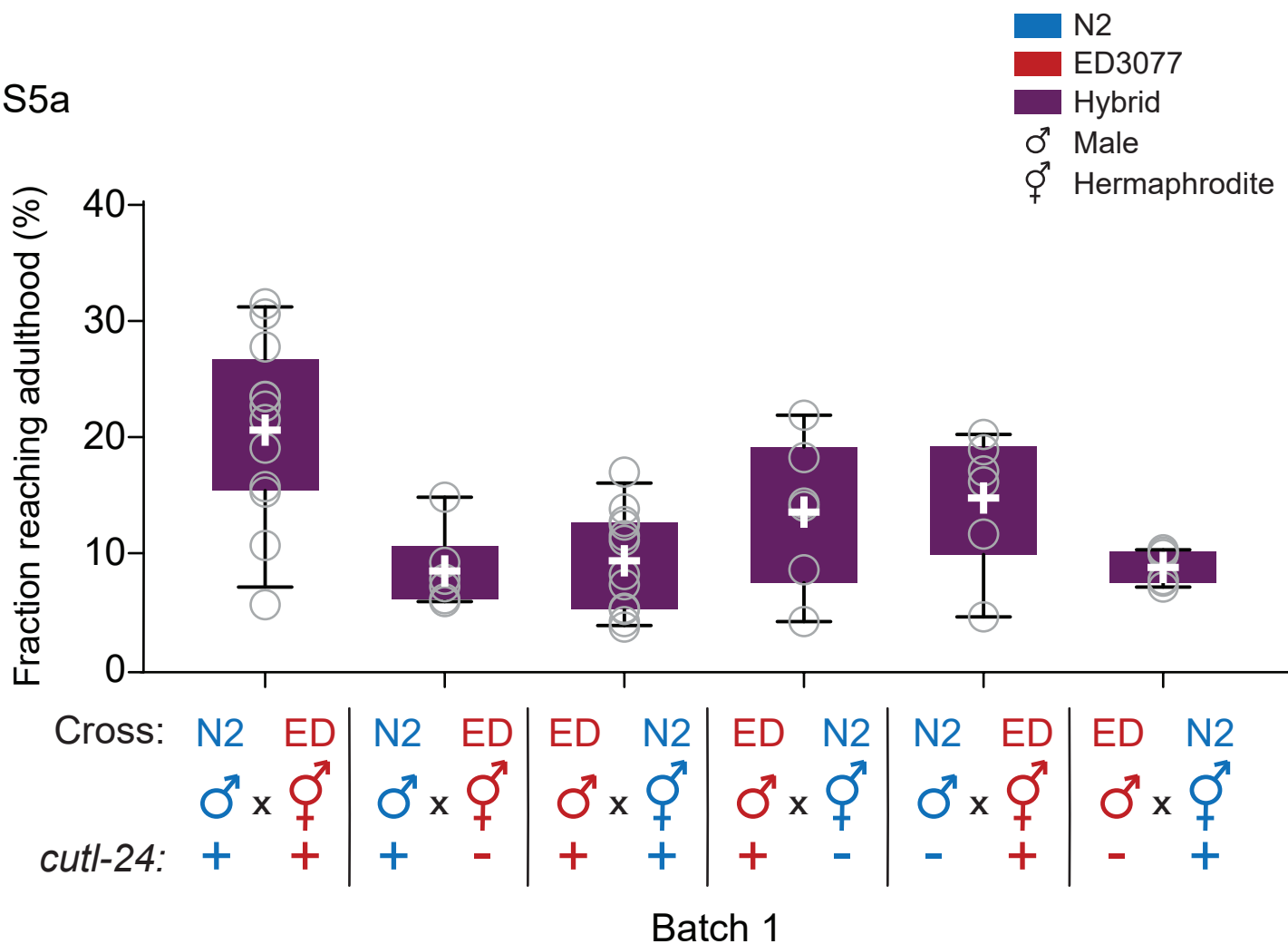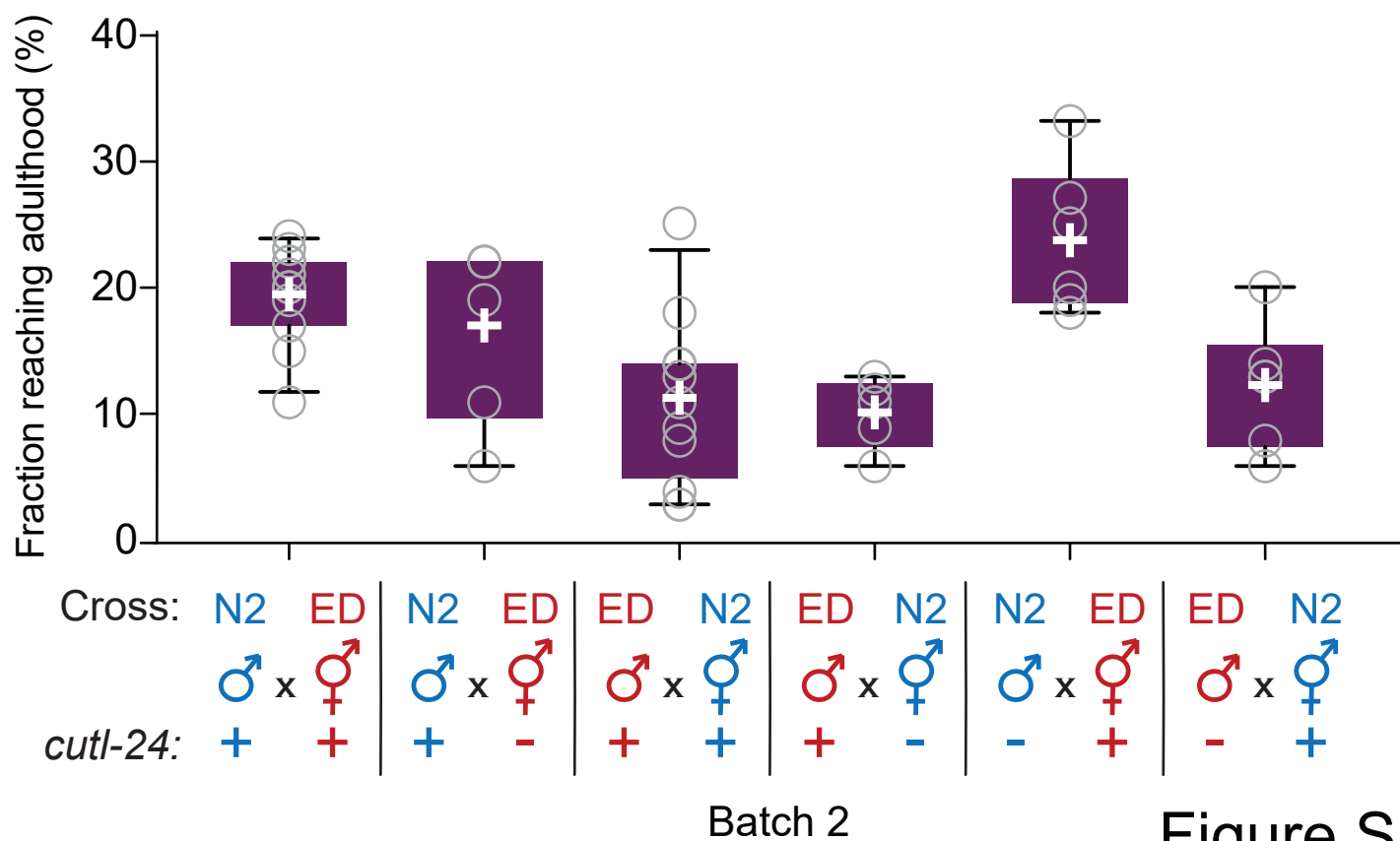

Figure S5

S5b

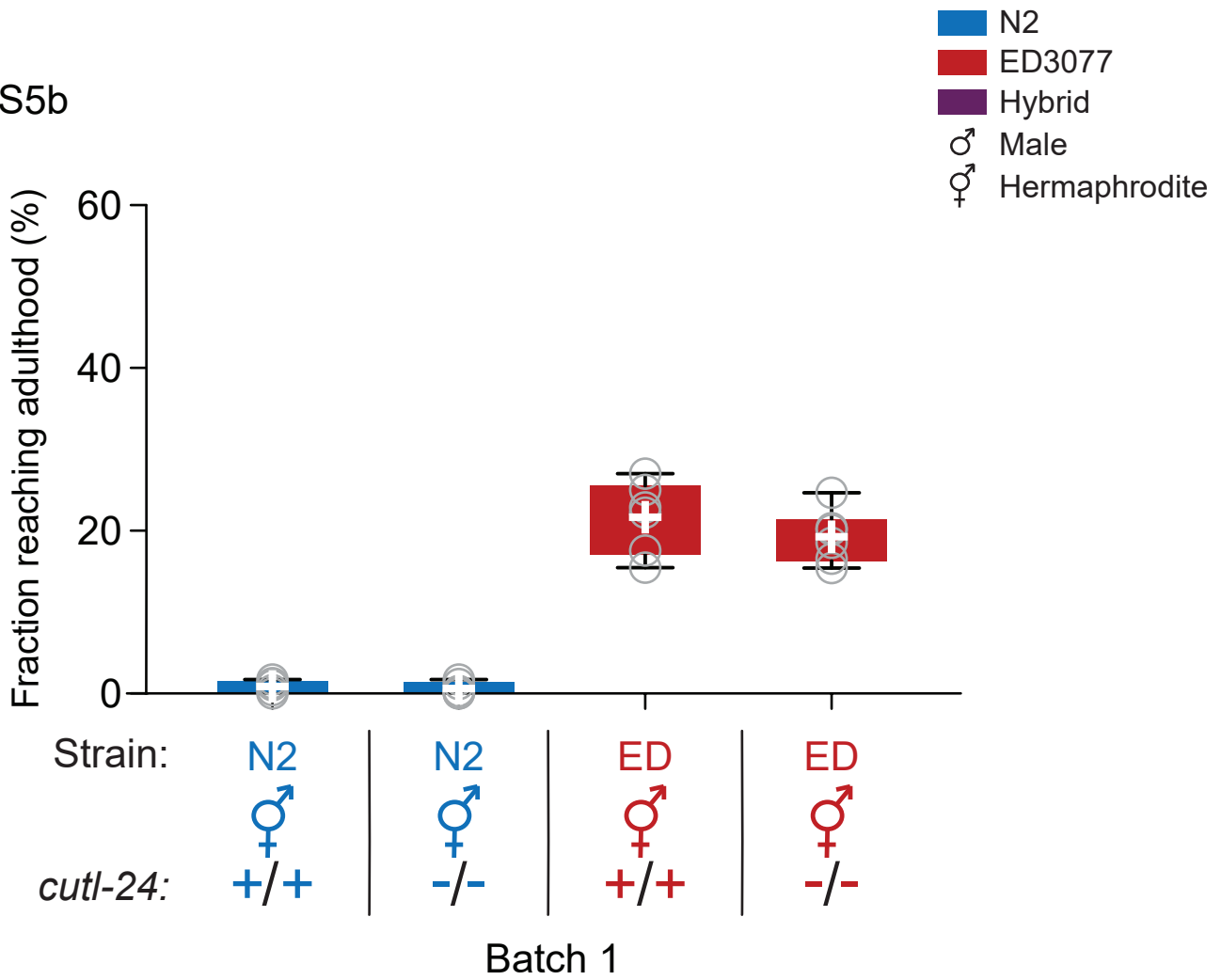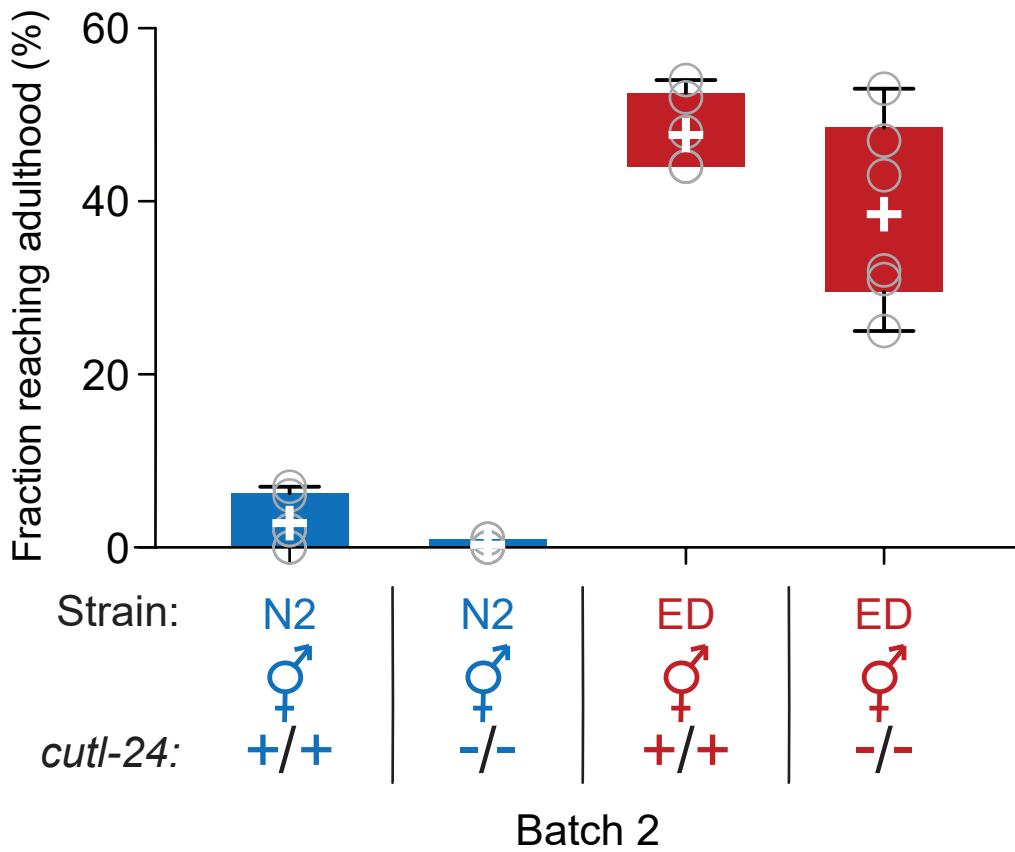

Supplement: Supplementary file 5 — Additional file 5: Supplementary Figure 5. cutl-24 in ED3077 mothers is required for tunicamycin resistance in their inter-strain hybrid progeny. Data are as in Fig. 5 of the main text, except that all measures are reported as raw proportions without normalization and are reported for two experimental batches performed 6 months apart. [file 12864_2022_9063_MOESM5_ESM.pdf]
