# Supplementary material for: A role for worm cutl-24 in background- and parent-of-origin-dependent ER stress resistance
Source: BMC Genomics. 2022 Dec 20;23:842. doi: 10.1186/s12864-022-09063-w (PMC9764823; doi:10.1186/s12864-022-09063-w)
Supplement: Supplementary file 4 — Additional file 4: Supplementary Figure 4. Top-scoring loci from RH-seq mapping of tunicamycin resistance. Data are as in Fig. 4 of the main text except that each panel reports results from the top 10 most significant genes from reciprocal hemizygosity tests for the impact of variation between ED3077 and N2 on development of their F1 hybrid in the presence of tunicamycin. [file 12864_2022_9063_MOESM4_ESM.pdf]

S4a

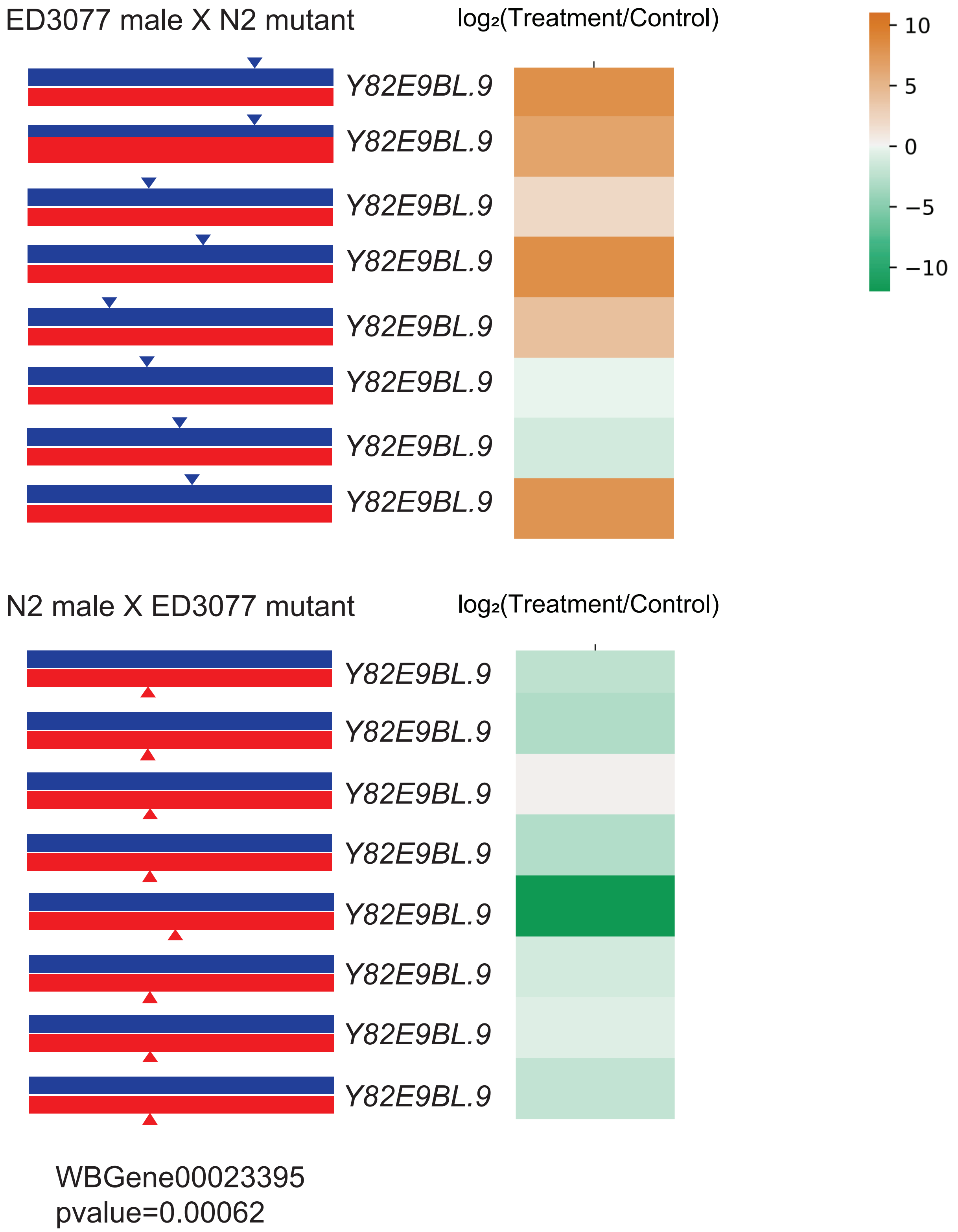

Figure S4

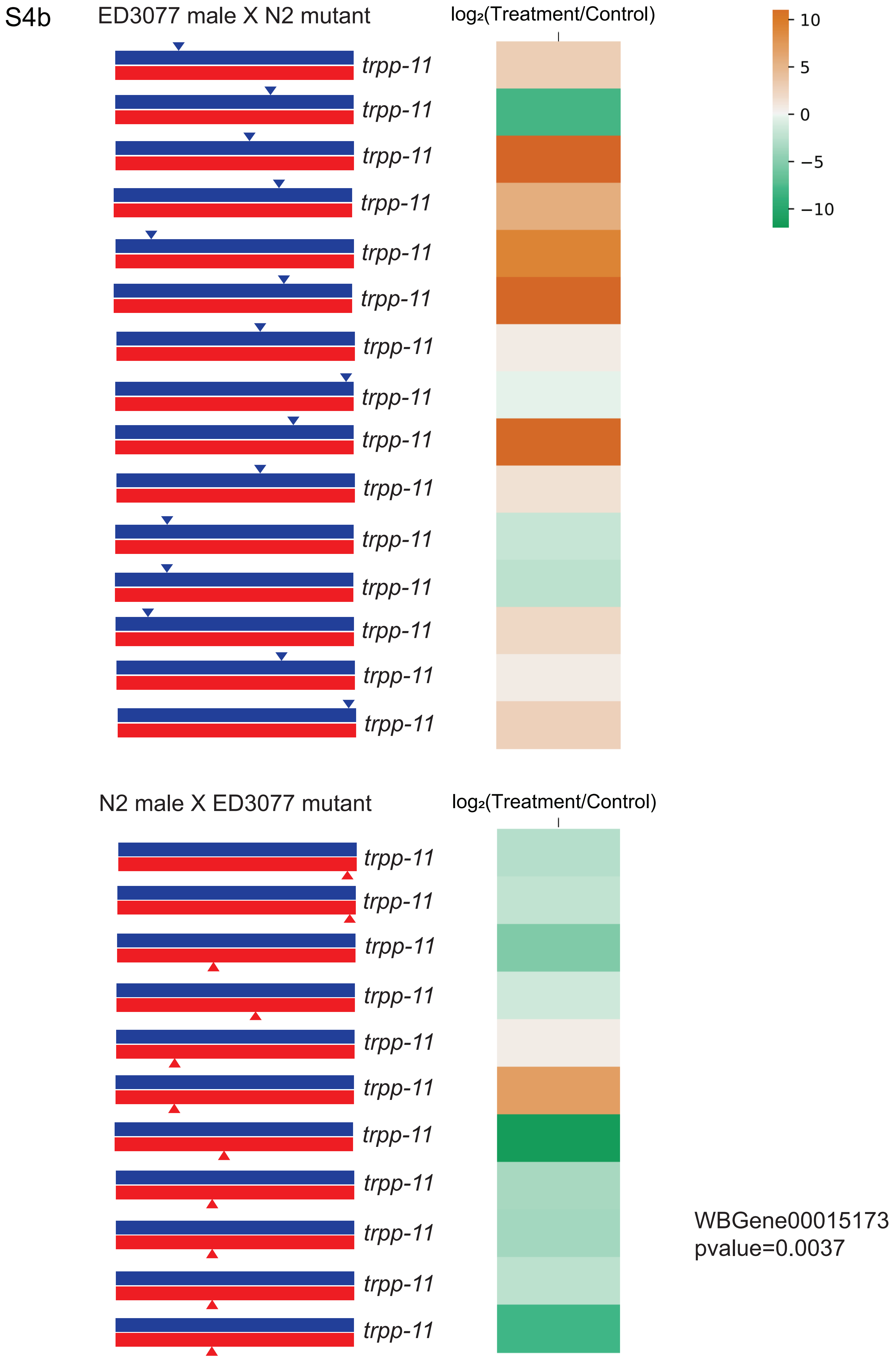
S4b
ED3077 male X N2 mutant
log2(Treatment/Control)
trpp-11
10
5
0
−5
−10
N2 male X ED3077 mutant
log2(Treatment/Control)
WBGene00015173
pvalue=0.0037

S4c

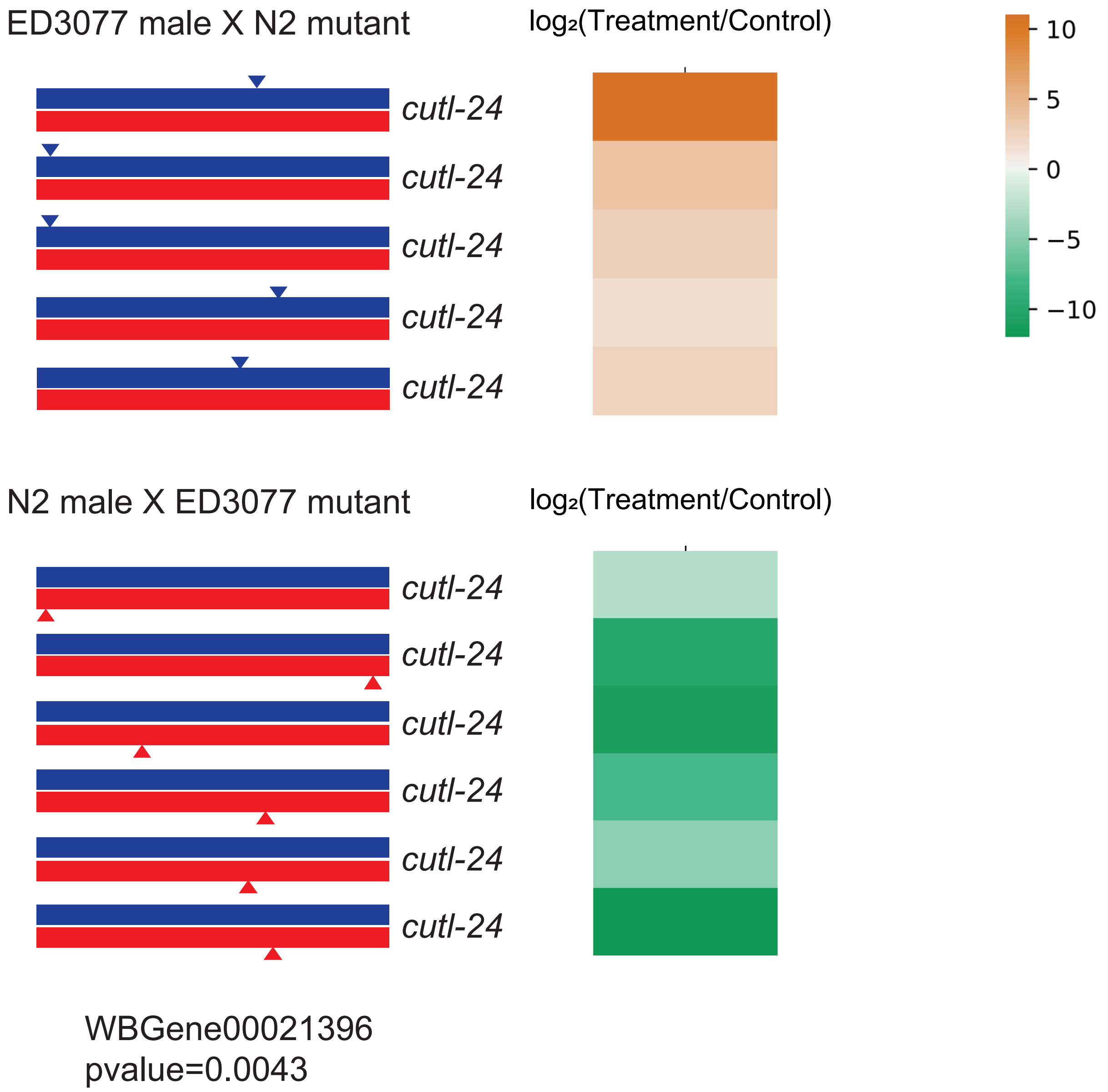

ED3077 male X N2 mutant
log2(Treatment/Control)
cutl-24
cutl-24
cutl-24
cutl-24
cutl-24
10
5
0
−5
−10
N2 male X ED3077 mutant
log2(Treatment/Control)
cutl-24
cutl-24
cutl-24
cutl-24
cutl-24
cutl-24
WBGene00021396
pvalue=0.0043

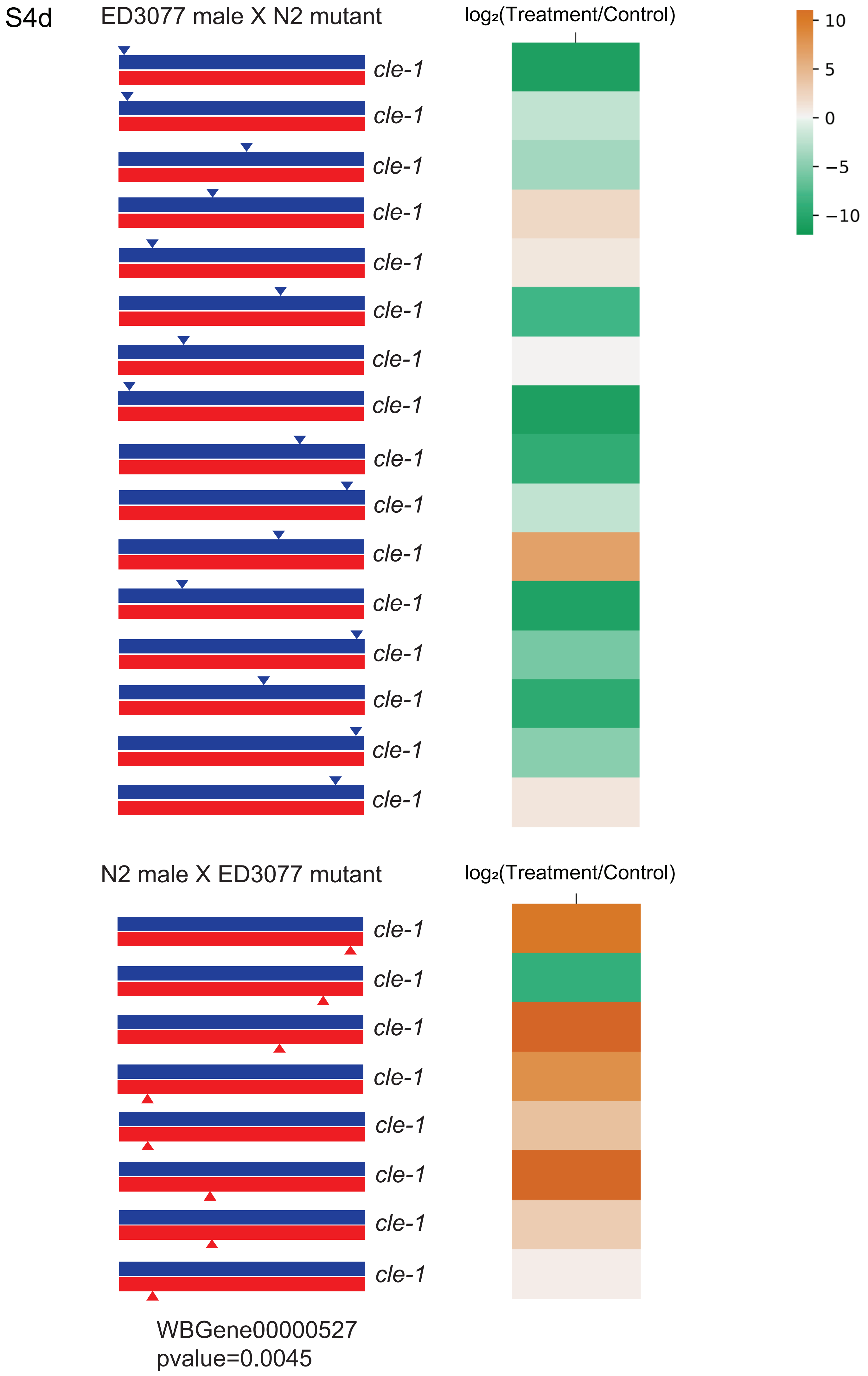
S4d
ED3077 male X N2 mutant
log₂(Treatment/Control)
10
5
0
−5
−10
cle-1
N2 male X ED3077 mutant
log₂(Treatment/Control)
WBGene00000527
pvalue=0.0045

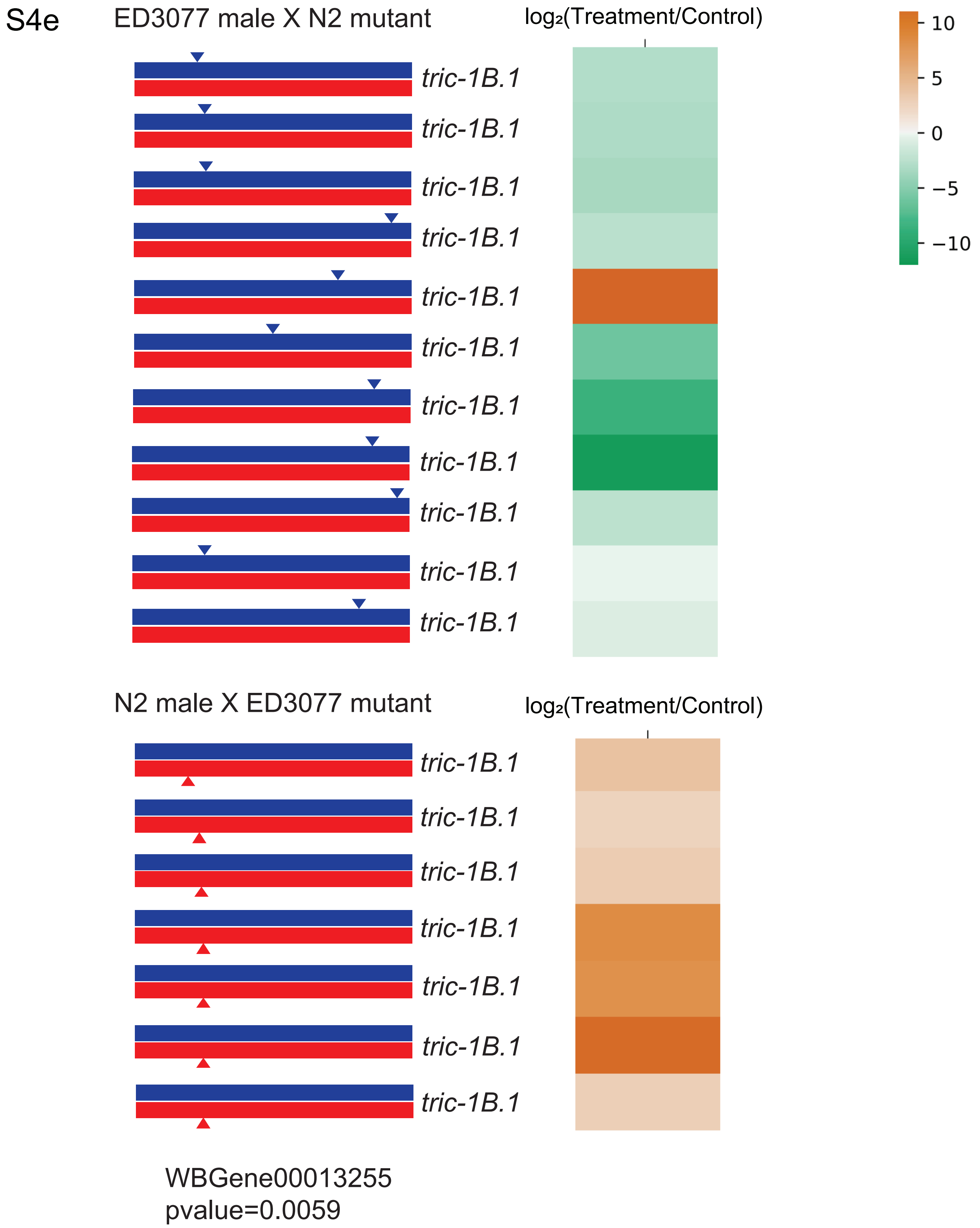
S4e
ED3077 male X N2 mutant
log₂(Treatment/Control)
10
5
0
−5
−10
tric-1B.1
tric-1B.1
tric-1B.1
tric-1B.1
tric-1B.1
tric-1B.1
tric-1B.1
tric-1B.1
tric-1B.1
tric-1B.1
tric-1B.1
N2 male X ED3077 mutant
log₂(Treatment/Control)
tric-1B.1
tric-1B.1
tric-1B.1
tric-1B.1
tric-1B.1
tric-1B.1
tric-1B.1
WBGene00013255
pvalue=0.0059

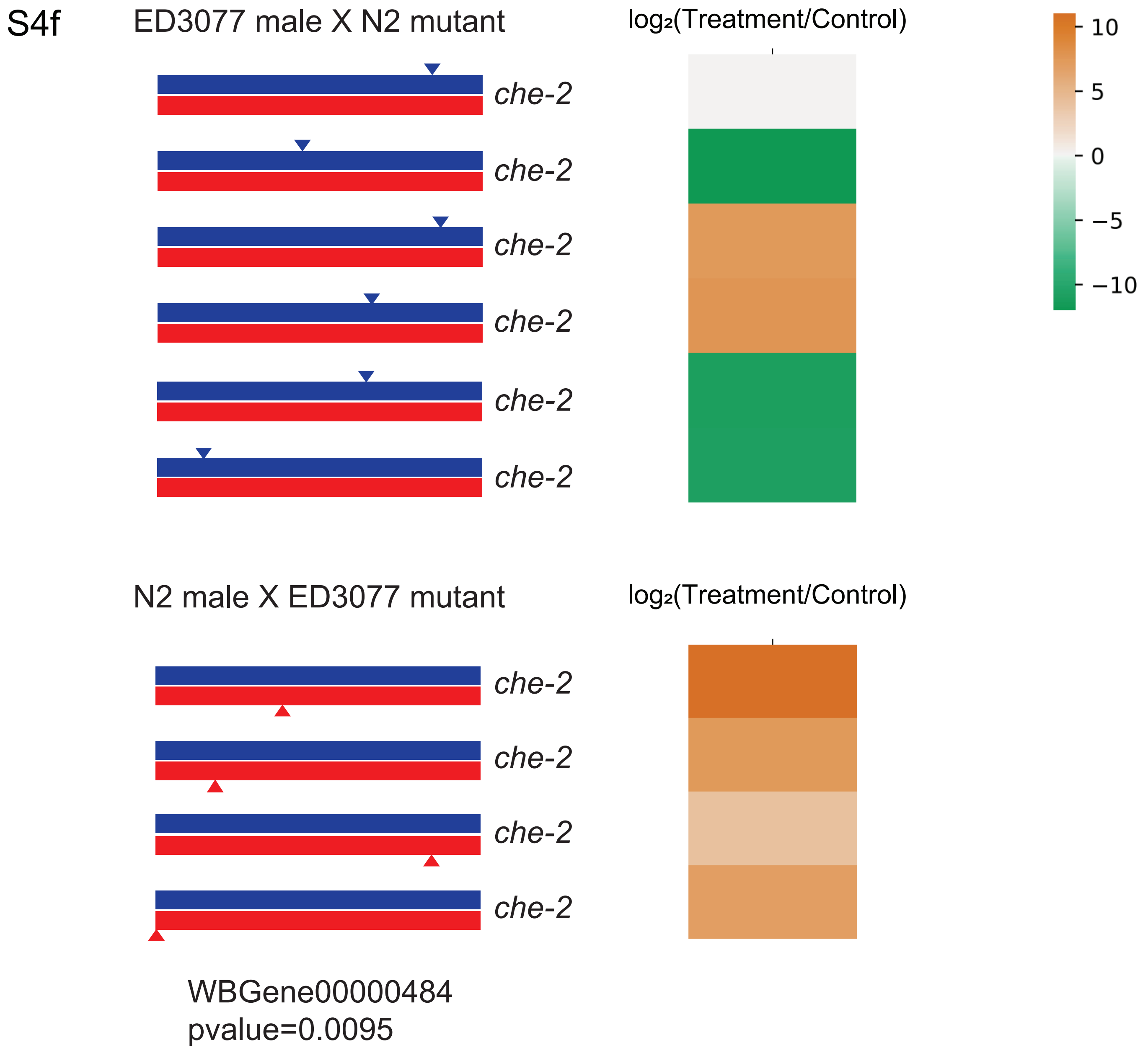
S4f
ED3077 male X N2 mutant
log2(Treatment/Control)
che-2
che-2
che-2
che-2
che-2
che-2
10
5
0
−5
−10
N2 male X ED3077 mutant
log2(Treatment/Control)
che-2
che-2
che-2
che-2
WBGene00000484
pvalue=0.0095

S4g

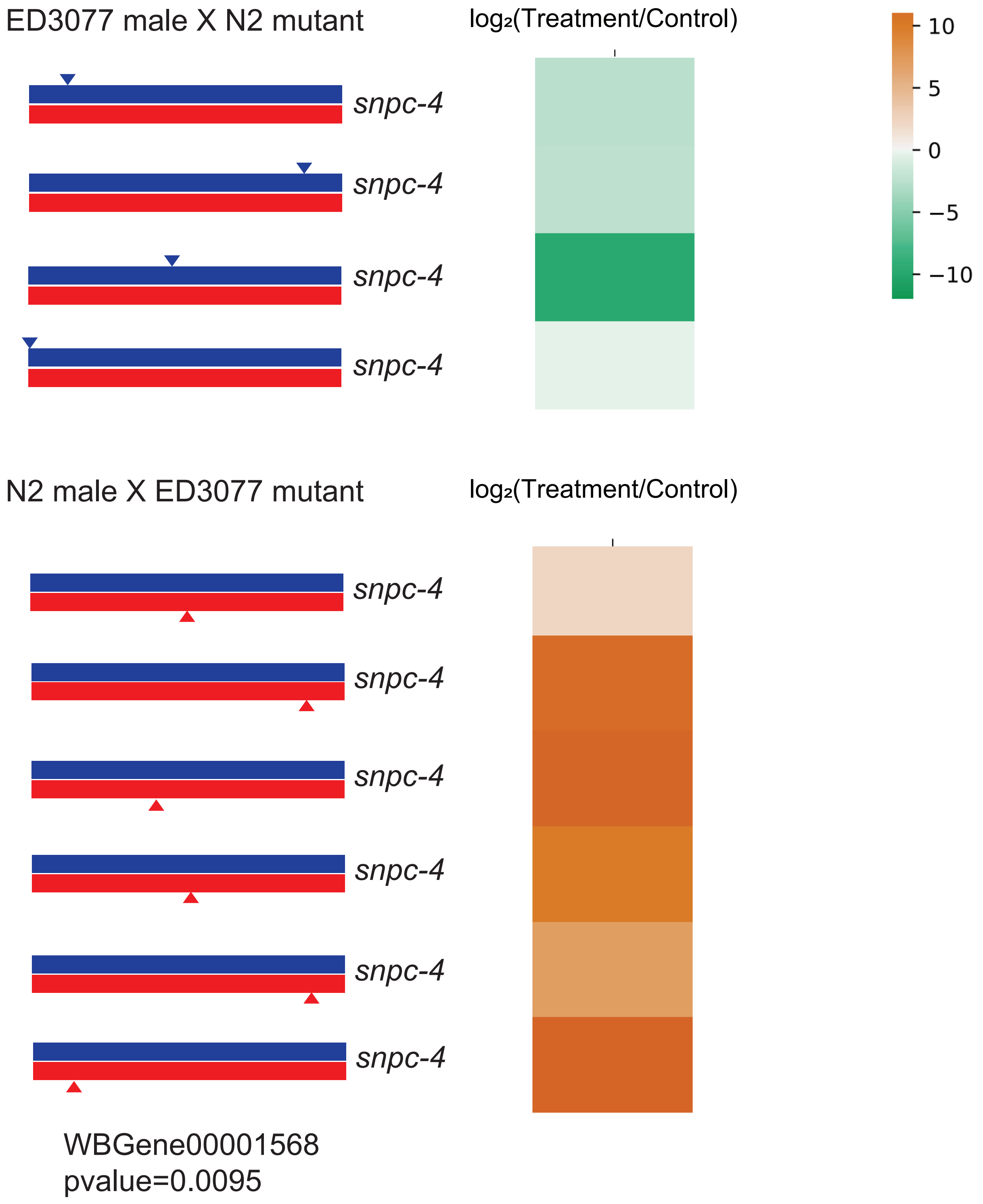
ED3077 male X N2 mutant
log₂(Treatment/Control)
snpc-4
snpc-4
snpc-4
snpc-4
10
5
0
−5
−10
N2 male X ED3077 mutant
log₂(Treatment/Control)
snpc-4
snpc-4
snpc-4
snpc-4
snpc-4
snpc-4
WBGene00001568
pvalue=0.0095

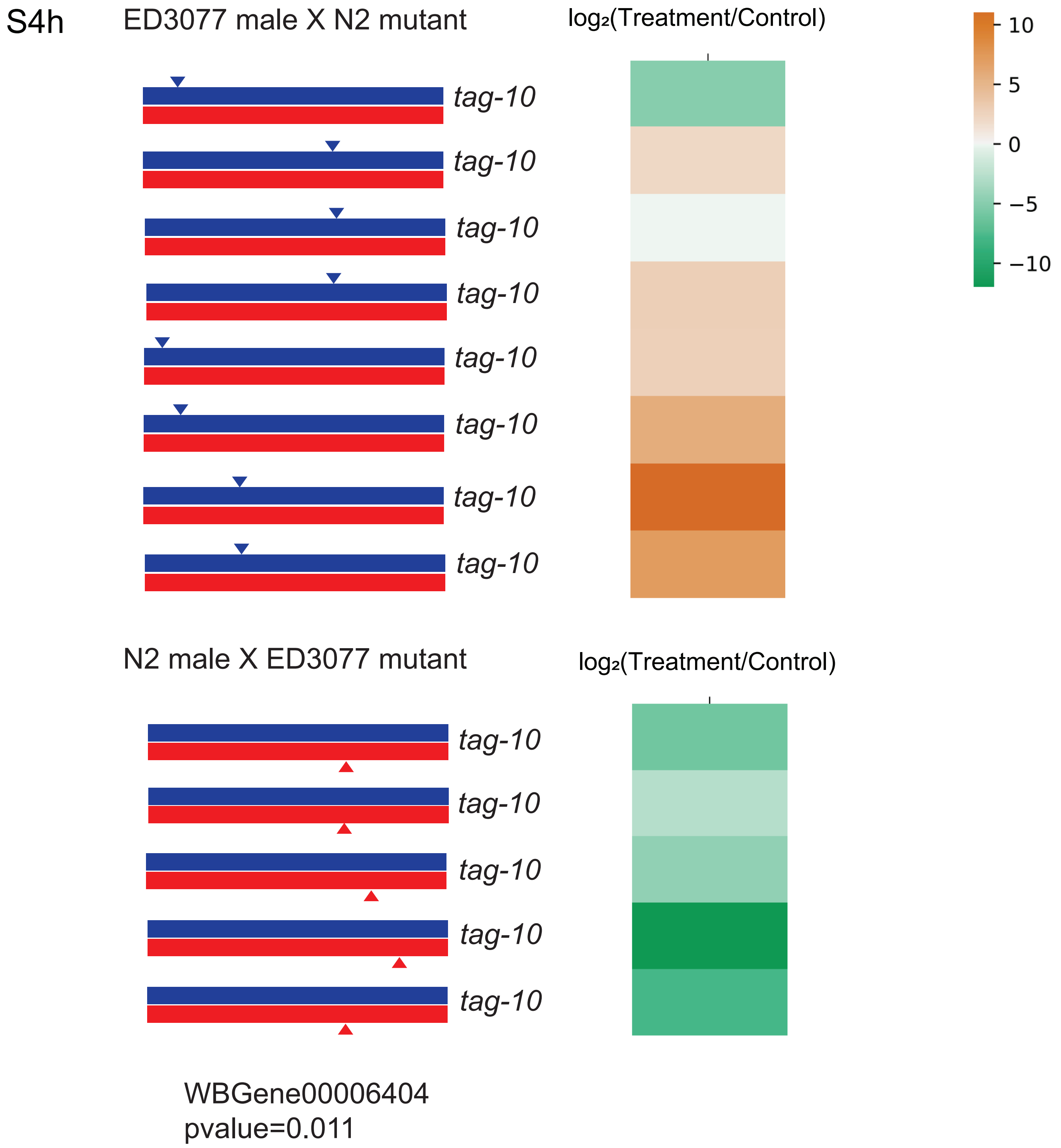

S4h
ED3077 male X N2 mutant
log2(Treatment/Control)
tag-10
tag-10
tag-10
tag-10
tag-10
tag-10
tag-10
tag-10
10
5
0
−5
−10
N2 male X ED3077 mutant
log2(Treatment/Control)
tag-10
tag-10
tag-10
tag-10
tag-10
WBGene00006404
pvalue=0.011

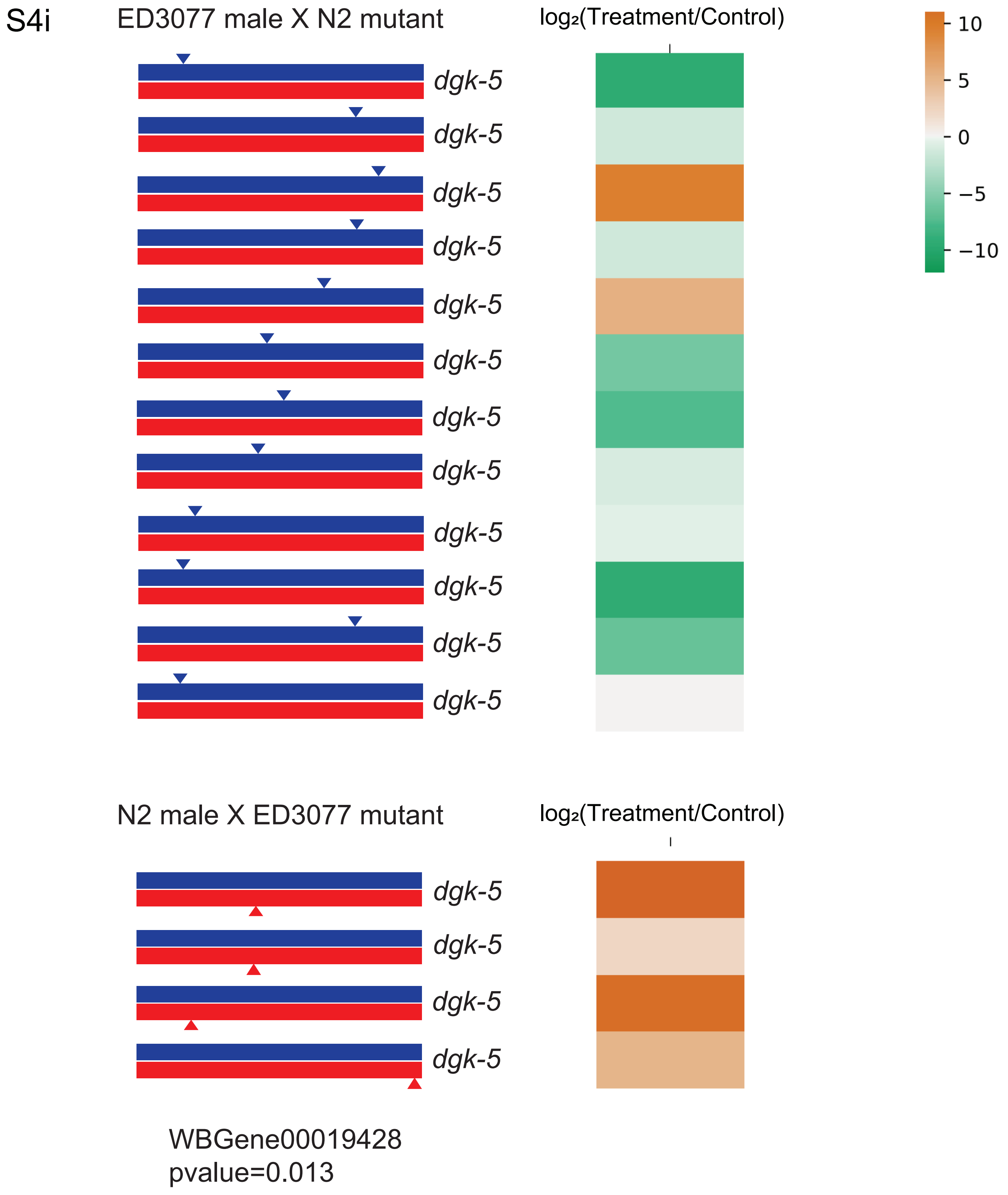
S4i
ED3077 male X N2 mutant
log2(Treatment/Control)
dgk-5
dgk-5
dgk-5
dgk-5
dgk-5
dgk-5
dgk-5
dgk-5
dgk-5
dgk-5
dgk-5
dgk-5
10
5
0
−5
−10
N2 male X ED3077 mutant
log2(Treatment/Control)
dgk-5
dgk-5
dgk-5
dgk-5
WBGene00019428
pvalue=0.013

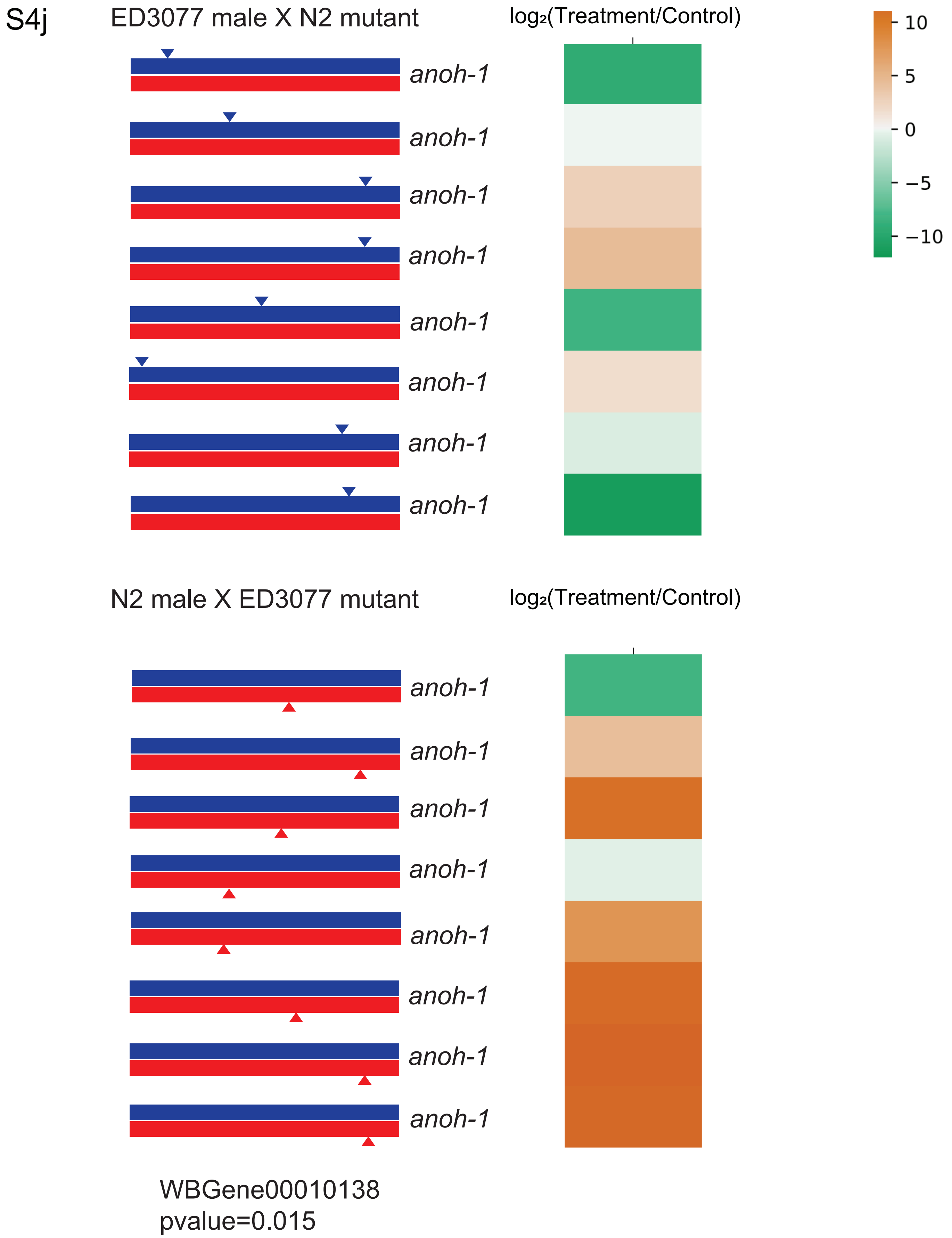
S4j
ED3077 male X N2 mutant
log₂(Treatment/Control)
anoh-1
anoh-1
anoh-1
anoh-1
anoh-1
anoh-1
anoh-1
anoh-1
10
5
0
−5
−10
N2 male X ED3077 mutant
log₂(Treatment/Control)
anoh-1
anoh-1
anoh-1
anoh-1
anoh-1
anoh-1
anoh-1
anoh-1
WBGene00010138
pvalue=0.015
